# A Rare Bacteremia in a Patient With Osteomyelitis and Sacral Ulcer: A Case Report of Tissierella praeacuta and Parvimonas micra

**DOI:** 10.7759/cureus.74314

**Published:** 2024-11-23

**Authors:** Jaskomal Phagoora, Brett Agrest, Moshe Kabariti, Sukhpreet Saini, Yanni Hedjar

**Affiliations:** 1 Medicine, Touro College of Osteopathic Medicine, New York, USA; 2 General Surgery, Brookdale University Hospital Medical Center, New York, USA

**Keywords:** anaerobic bacteremia, anaerobic osteomyelitis, clostridium hastiforme, emergency surgical debridement, geriatric infectious disease, parvimonas micra, rare anaerobic infections, sacral pressure ulcer, sacral ulcer complications, tissierella praeacuta

## Abstract

*Tissierella praeacuta* and *Parvimonas micra *are anaerobic bacteria rarely encountered in clinical practice, making their identification in bacteremia significant. These organisms are typically found in the human gut and oral flora and are generally considered low-virulence. However, in patients with compromised immunity or significant comorbidities, they can lead to severe infections, including bacteremia. Osteomyelitis, particularly when associated with chronic sacral ulcers, can increase the risk of secondary infections, complicating patient outcomes.

We present the case of an 86-year-old male patient with a complex medical history, including atrial fibrillation, congestive heart failure, and chronic obstructive pulmonary disease, who developed bacteremia caused by *T. praeacuta* and *P. micra*. The patient was admitted with a severely infected stage IV sacral ulcer, which had progressed rapidly and was accompanied by systemic signs of sepsis. Despite broad-spectrum antibiotic therapy and surgical debridement, the patient’s condition worsened, leading to the identification of rare pathogens in blood cultures. Subsequent management included adjusting antimicrobial therapy and aggressive wound care, ultimately stabilizing the patient’s condition.

This case highlights the significance of recognizing and accurately diagnosing rare anaerobic bacteremia in patients with chronic ulcers and osteomyelitis. The involvement of *T. praeacuta* and *P. micra*, typically low-virulence organisms, in such severe infections underscores the need for thorough microbiological evaluation and a multidisciplinary approach to treatment. Early identification and appropriate management are crucial in preventing adverse outcomes in complex cases such as this.

## Introduction

*Tissierella praeacuta* (also called Clostridium hastiforme) and *Parvimonas micra* are both anaerobic bacteria rarely encountered in clinical practice, making their identification and associated bacteremia noteworthy. *T. praeacuta *is a gram-positive anaerobic rod, while *P. micra* is a gram-positive anaerobic coccus. Tissierella and Parvimonas are found in the human gut and oral flora respectively and are generally considered low-virulence organisms. However, under certain conditions, such as in patients with compromised immunity or significant comorbidities, they can cause severe infections, including bacteremia [1,2].

Osteomyelitis is an infection of the bone, typically caused by bacteria. When associated with a sacral ulcer, which is often a chronic pressure ulcer located near the sacrum, the risk of secondary infections, including bacteremia, significantly increases. Osteomyelitis commonly presents with localized pain, tenderness, swelling, and, in chronic cases, the formation of draining sinus tracts. When complicated by bacteremia, patients may exhibit systemic symptoms such as fever, chills, and malaise [3]. 

The progression of bacteremia in the context of osteomyelitis can lead to sepsis, a life-threatening systemic response to infection, especially if the causative organisms are atypical or resistant to standard treatments. The rarity of bacteremia caused by *T. praeacuta* and *P. micra* underscores the importance of early recognition, accurate microbiological diagnosis, and appropriate management to prevent severe outcomes [1,2]. In this case report, we present a patient with osteomyelitis secondary to a chronic sacral ulcer who developed bacteremia caused by *T. praeacuta* and *P. micra*.

## Case presentation

An 86-year-old male patient with a complex medical history, including atrial fibrillation on anticoagulation (apixaban), congestive heart failure, chronic obstructive pulmonary disease, coronary artery disease, and hypertension, presented to the emergency department via emergency medical services (EMS). EMS was summoned by the patient's daughter-in-law due to concerns about sacral decubitus ulcers, which were reportedly recent but had rapidly progressed. 

Upon arrival at the patient’s residence, EMS noted a strong odor emanating from the ulcers, which were extensive and appeared to be severely infected based on clinical presentation. The patient was hypotensive and tachycardic, prompting immediate transfer to the crash unit for suspected septic shock. The patient appeared malnourished and in poor general condition. The physical examination revealed significant findings, including poor oral hygiene with white, scale-like lesions on the tongue and gums, bilateral lower extremity pitting edema (2+ to the level of the knee), and multiple severe pressure ulcers. The most concerning was a 15 cm stage IV sacral ulcer with purulent and bloody discharge, extending to the bone. The lower legs were contracted, and the patient was unable to fully extend his extremities. An additional unstageable, erythematous ulcer on the foot was also noted, which was tender to palpation.

Given the severity of the presentation, a comprehensive diagnostic workup was initiated, including blood cultures, complete blood count, comprehensive metabolic panel, coagulation studies, lactic acid, creatine phosphokinase, troponins, chest X-ray, and urinalysis. Piperacillin-tazobactam was started, and the general surgery team was consulted for potential debridement and evaluation for necrotizing fasciitis. The patient was subsequently taken to the operating room for debridement of the sacral ulcer.

Intraoperatively, a large area of necrotic tissue measuring 18 x 10 x 7 cm was debrided, including the right buttock ulcer, which extended to the bone. A bone biopsy and culture of the right ischium were obtained, and significant undermining of the wound was noted, extending to the posterior aspect of the right thigh. The wound was classified as Class IV. Blood cultures were also obtained during the procedure. On Day 2, the patient was transferred to the Surgical Intensive Care Unit (SICU) for close monitoring. Although he remained afebrile, a leukocytosis of 20.8 was noted. Past wound cultures had yielded Pseudomonas, Proteus, and Escherichia coli. Empiric antibiotic therapy with piperacillin-tazobactam was continued, with dosing adjustments as recommended by the Infectious Disease (ID) team. Urine culture from this day grew 75,000 CFU/mL of oxidase-negative gram-negative bacilli, while blood and wound cultures remained pending.

**Figure 1:**
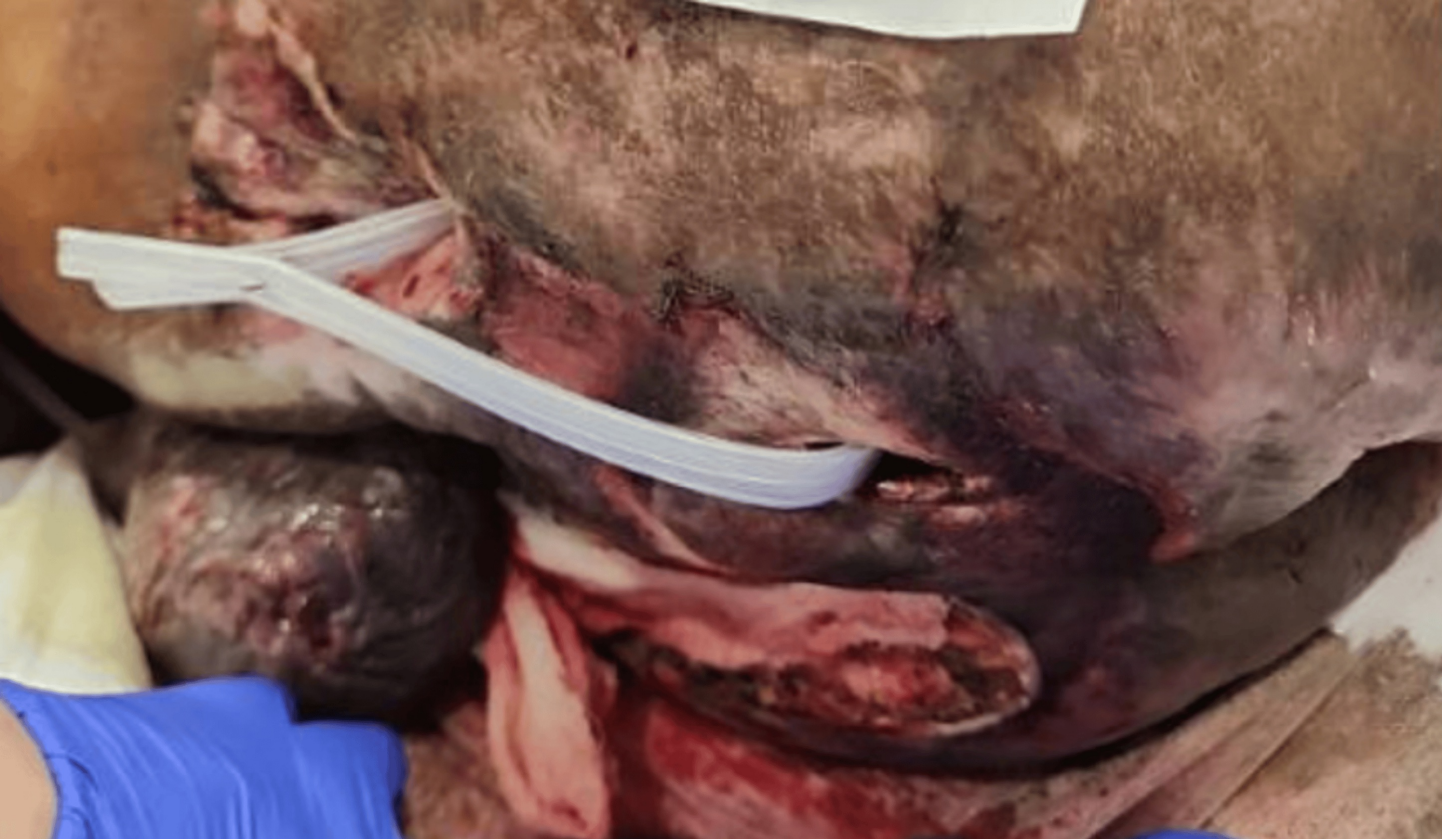
Pressure ulcer with a Braden scale score of 12

On Day 3, skin integrity notes show a pressure ulcer with a Braden scale score of 12. Figure 1 shows pressure ulcers on that day.

By Day 4, the urine culture results indicated the presence of Pseudomonas aeruginosa, and the wound culture revealed a rare growth of Group B Streptococcus. The ID team recommended continuing with piperacillin-tazobactam until further culture results could guide therapy adjustments.

On Day 10, despite ongoing treatment, concerns about persistent infection led to the de-escalation of piperacillin-tazobactam to ceftriaxone and metronidazole, as recommended by ID. If the blood cultures show a new organism, the regimen can be altered as needed. 

Day 11 marked a significant turning point in the patient's clinical course. He was transferred back to the SICU due to worsening hemodynamic instability, including hypotension and tachycardia. Blood cultures from day 1 returned with gram-negative rods. Anaerobic blood cultures were taken on this day. This bacteremia, coupled with the patient's deteriorating condition, necessitated the initiation of piperacillin-tazobactam. Ultrasound revealed gallbladder distention with cholelithiasis and thickening of the gallbladder wall, raising concerns for cholecystitis.

Further investigations on Day 13 through ultrasound and magnetic resonance cholangiopancreatography identified acute cholecystitis and diffuse dilation of the common bile duct and pancreatic duct. The patient was subsequently scheduled for endoscopic retrograde cholangiopancreatography (ERCP) with endoscopic ultrasound (EUS).

By Day 19, the patient underwent a successful ERCP with sphincterotomy and stone extraction, which alleviated the biliary obstruction. Subsequent days showed stabilization in the patient's clinical parameters, including normal amylase and lipase levels by Day 20. Anaerobic blood cultures from this day returned with rare pathogens: *Tissierella praeacuta* and *Parvimonas micra*.

By Day 22, blood cultures were negative, and the patient was maintained on metronidazole and daptomycin was added as recommended by ID.

By Day 30, the patient was medically stable, with no acute events or distress in the days leading up to discharge. He was cleared for transfer to a nursing home for rehabilitation and wound care, pending bed availability as coordinated by social work.

Table 1 describes the timeline of different blood markers in the patient.

**Table 1:** Timeline of blood samples WBC: White blood cell (cells per microliter (µL)), HGB: Hemoglobin (g/dL), INR: International normalized ratio (a measure of blood coagulation), Na: Sodium (mEq/L), Creatine (mg/dL): Creatinine (a measure of kidney function), Glu (mg/dL): Glucose, ALB (g/dL): Albumin, AST (U/L): Aspartate aminotransferase (a liver enzyme), ALT (U/L): Alanine aminotransferase (another liver enzyme), ALP (U/L): Alkaline phosphatase (an enzyme related to the liver and bones), Bilirubin (mg/dL): A substance produced by the breakdown of red blood cells, measured to assess liver function and diagnose jaundice.

Day	WBC (cells per (µL))	HGB (g/dL)	INR	Na (mEq/L)	Creatine (mg/dL)	Glu (mg/dL)	ALB (g/dL)	AST (U/L)	ALP (U/L)	Total Bilirubin (mg/dL)
Reference Range	4.0–11.0 x 10³ cells/µL	Female: 12.0–16.0 Male: 13.5–17.5	0.8–1.2	135–145	0.6–1.2	70–100	3.5–5.0	10–40	44–147	0.1–1.2
1	20.8	11.2	1.34	133	1.5	134	2.4	52	188	1.9
2	16.8	9.7	1.48	135	-	142	1.7	38	121	-
3	16.1	8.2	1.42	137	-	97	2.2	38	93	-
4	14.4	9	1.25	137	-	127	2.1	50	124	-
5	11.6	10.3	1.11	137	-	123	2.1	53	153	-
6	12.6	9.2	-	133	0.6	76	1.9	60	140	0.9
7	11.4	9.4	-	137	0.6	77	2	32	166	0.8
8	12.2	9.4	-	134	0.6	84	2	31	169	0.7
9	9.8	9.3	-	133	0.5	87	2	26	160	0.9
10	8.1	9.9	-	133	0.5	88	2.1	23	160	0.7
11	16.2	9	2.08	130	0.7	108	2	33	174	3.5
12	7.6	9.1	1.67	131		113	2	36	175	-
13	5.7	8.7	1.41	130		122	1.9	49	195	-
14	4.7	9	-	134	0.5	90	1.9	57	203	4
15	4.3	8.8	-	134	0.5	105	1.8	60	217	3.7
16	5.8	8.5	-	134	0.5	134	1.8	63	224	3.3
17	5.5	8.8	1.4	134	0.4	90	1.8	62	234	3
18	4.8	8.9	1.35	132	0.4	86	1.8	62	232	2.8
19	4.2	8.8	1.39	133	0.4	162	1.9	71	231	2.3
23	6.2	9.3	1.4	133	0.3	98	1.9	89	426	1.9
24	6	10	1.23	132	0.4	96	2.1	75	427	2
25	4.7	10	1.23	134	0.4	83	2.1	78	463	2.1
26	5.1	9.9	-	132	0.5	90	2.1	90	491	2.1
27	5	10.1	-	134	0.5	94	2.2	82	452	2.3
28	6.1	9.4	-	133	0.6	86	2.2	107	512	2
29	3.6	10.5	-	136	0.4	71	2.3	128	530	1.9

## Discussion

**Figure 2:**
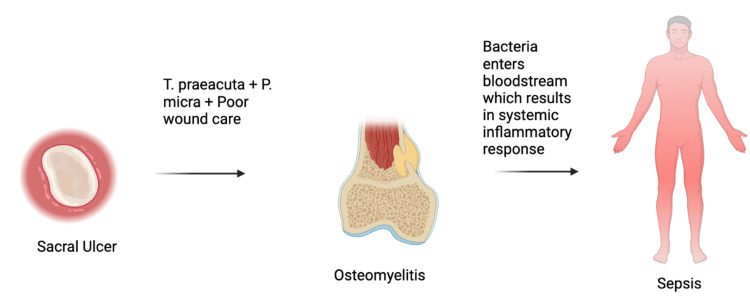
Pathological progression to sepsis The figure illustrates the pathological progression to sepsis, conceptualized based on the case of Tissierella praeacuta bacteremia as reported in reference [1]. Created with Biorender.com

The development of bacteremia in the setting of osteomyelitis can progress to sepsis, a potentially fatal systemic reaction to infection, particularly when the causative organisms are unusual. This progression is depicted in Figure 2.

*T. praeacuta* is an obligate-anaerobic gram-variable bacillus commonly isolated in cultures of human gastrointestinal microbiota and soil [1,4]. It is one of the five species that belong to the Tissierella genus and was originally identified in fecal samples taken from infants in 1908 by Tissier [5,6]. This bacterium is the only species from the genus that is pathogenic in human samples [7]. Given this, *T. praeacuta* is still a relatively rare culprit in disease processes, with only a handful of cases scattered throughout the literature. The bacterium has been isolated in various pathologies, including but not limited to septic pseudoarthrosis of the knee joint, bacteremia secondary to pyonephrosis, bacteremia secondary to osteomyelitis of the foot, bacteremia secondary to osteomyelitis of the tibia, and infectious thrombophlebitis of the left internal jugular vein secondary to sphenoid sinusitis [1,4-6]. While all of these case reports give insight into the pathogenesis of *T. praeacuta* infections, of particular interest is a case report of a 45-year-old male patient with bacteremia secondary to chronic sacral and ischial decubitus ulcers. The patient was reported to have been admitted twice for complications related to the ulcers, with *T. praeacuta* being identified in blood cultures in both instances. The bacterium was not found in the culture of the wound itself; however, the researchers indicated the ulcers as a likely source of the Tissierella bacteremia [7].

The case presented in this current report has notable overlap with the aforementioned report, with one major caveat being that *T. praeacuta* was cultured within the actual wound itself. This presentation provides insight into the question put forth by the authors of the previous report regarding the prevalence of *T. praeacuta* in human infections. They suggested that although the bacterium may indeed be a rare cause of infection, it also may be a common cause that is not routinely tested for. This explanation is plausible, considering that the required mechanism of identification of *T. praeacuta* includes analysis with the BACT/ALERT blood culture system using MALDI-TOF MS [4,7]. Additionally, *T. praeacuta* is susceptible to beta-lactams, rifampin, metronidazole and other antibiotics commonly utilized to treat other bacterial infections, which means that less importance is placed on its identification in a clinical setting [7]. With this in mind, this current case report elicits greater suspicion that *T. praeacuta* may be a more common source of infection than previously thought.

*P. micra* is a Gram-positive obligate anaerobe commonly located in the gastrointestinal and upper respiratory tracts of humans. It is known to cause infections linked to periodontal disease, alveolar or peritonsillar abscesses, chronic upper respiratory conditions like chronic sinusitis or otitis media, and pyogenic pulmonary diseases [8]. Additionally, *P. micra* has been associated with deeper infections, including those around artificial joints. Notably, individuals with lower socioeconomic status are more susceptible to this infection, potentially due to challenges in accessing routine preventive dental care [2].

This case of *P. micra* is unique and differs from other recent case reports due to multiple reasons. While *P. micra *infections have been documented in severe cases of pneumonia, bacteremia, and mono-bacterial infections primarily affecting internal organs, the involvement of large necrotic pressure ulcers in this patient, gives an additional dimension to the infection [9]. The extensive sacral ulcer, measuring 15 cm and extending to the bone, combined with the need for aggressive surgical debridement, highlights a level of tissue involvement not typically associated with P. micra infections, which are often more localized [10].

Additionally, the severe systemic deterioration observed in this patient, including multi-organ involvement with concurrent acute cholecystitis which required an ERCP, further distinguishes this case from others. In other case reports, *P. micra* infections generally remained within a single system and did not escalate [2].

The rapid clinical decline, as shown by the transition from the initial presentation to critical care such as SICU, and the emergence of bacteremia with both *P. micra *and* T. praeacuta*, highlights the severity and rarity of this case. Unlike the cases reported in the literature, which often detail infections in areas such as the lungs or oral cavity, this case involved a broader systemic impact, needing intensive surgical intervention and a comprehensive approach to treatment [2].

This case contributes significantly to the existing literature by highlighting the bacterium's potential to cause life-threatening, multi-system infections in elderly, immunocompromised patients with complex medical histories. The case emphasizes the importance of a multidisciplinary approach in managing such infections. The presence of two rare pathogens with severe extensive tissue necrosis makes this case particularly challenging [11].

This case highlights the unusual presentation of bacteremia caused by *T. praeacuta* and *P. micra* in a patient with osteomyelitis secondary to a chronic sacral ulcer. Both bacteria are rarely encountered in clinical practice and are typically associated with low virulence, making their presence in this case particularly noteworthy. The identification of these pathogens underscores the importance of thorough microbiological evaluation, especially in patients with significant comorbidities and complex infections [12].

The ambiguity in this case centers around the source of bacteremia. While the chronic sacral ulcer and associated osteomyelitis are the most likely sources, the concurrent discovery of cholecystitis and biliary obstruction complicates the clinical picture. It is plausible that the bacteremia could have originated from the gastrointestinal tract, as both organisms are part of the gut flora [13]. This ambiguity underscores the challenges in managing complex infections in patients with multiple comorbidities and highlights the need for a multidisciplinary approach to treatment.

Early and aggressive wound management is also essential, especially in cases with severe necrotic tissue. This includes regular surgical debridement to remove dead tissue and prevent the spread of infection, combined with advanced wound care techniques such as negative pressure wound therapy to promote healing. Gelbard et al. demonstrated this by comparing a mortality rate of 14% in the early debridement group vs 25.8% in the late debridement group [14]. The use of hyperbaric oxygen therapy can also be helpful since it has been shown to enhance wound healing and fight anaerobic infections such as those caused by *P. micra* [10].

Wong et al. conducted a review on the use of antibiotics in cases of osteomyelitis complicated by sacral pressure ulcers. They found no evidence supporting the benefit of antibiotic use without concomitant wound debridement and closure. They recommend that antibiotic use be paired with debridement and closure to warrant its use [15]. The best medium for culture, whether it is an orthopedic bone biopsy or bedside cultures, depends on the particular organism of interest and patient risk factors. If a patient has a risk of bleeding disorders or severe comorbidities, they are less likely to undergo a bone biopsy. If a patient has a particular obligate anaerobe, they are more likely to have a bone biopsy cultured on thioglycolate broth [16].

Typically, bacteria found in sacral ulcers include *Staphylococcus aureus, Pseudomonas aeruginosa, Enterococcus faecalis, Proteus spp., *and* Bacteroides spp*. [17,18]. Due to the polymicrobial nature of these ulcers, cultures may not be helpful; one study found that positive cultures yielded a similar amount of bacteria in patients with osteomyelitis and those without. Furthermore, the same study found no association between treatment failure and culture positivity [19]. The mainstay of treatment for stage 4 decubitus ulcers should be surgical debridement of the wound.

## Conclusions

In this case report, the significance lies not in the alteration of treatment outcomes due to antibiotic therapy but rather in identifying and understanding rare anaerobic bacteria that could potentially complicate the clinical course, especially in immunocompromised patients or those with multiple comorbidities. Despite broad-spectrum antibiotics, the patient’s condition initially worsened until these rare pathogens were identified, prompting an adjustment in management. This case underlines the importance of thorough microbiological evaluation and a multidisciplinary approach to recognize unusual organisms that may otherwise be overlooked. Documenting such cases enhances our knowledge base for managing rare infections, emphasizing early identification and appropriate treatment strategies in complex patient scenarios.
